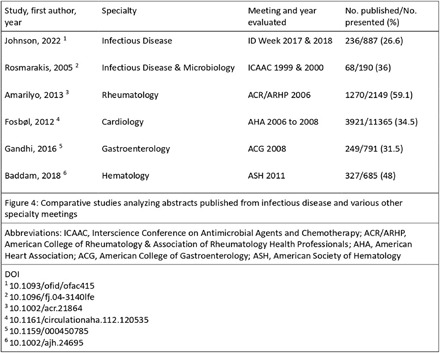# From Abstract to Article: Publication Rates of Abstracts Presented at the SHEA Spring Conference 2018 and 2021

**DOI:** 10.1017/ash.2025.227

**Published:** 2025-09-24

**Authors:** Aayushi Rajani, Shifa Karatela, Hitanshi Bhuptani, Purav Shah, Lipi Modha, Abhijeet Shukla, Juhi Amin, Justin Oring

**Affiliations:** 1 Medical College Baroda & Sir Sayajirao General Hospital; 2 Pandit Deendayal Upadhyay Medical College; 3 Mayo Clinic

## Abstract

**Background:** Conferences play a crucial role in the early dissemination of significant research to peers and experts within the same field. They provide a platform for receiving feedback, fostering collaborations, and refining groundbreaking findings, which can eventually be developed into full articles for publication in peer-reviewed journals. The transition of presented abstracts to full research journal publications is a key metric for evaluating research productivity, quality, and dissemination. Despite this, there is limited data on the proportion of abstracts that are ultimately published as full articles in peer-reviewed journals. **Method:** All abstracts (351) presented at the SHEA Spring Conference in 2018 and 2021 were indexed and cataloged from the 2018 online archive and the 2021 Antimicrobial Stewardship & Healthcare Epidemiology journal supplement. We then manually searched the top 20 results of both Google Scholar and PubMed to determine the publication status of each abstract as of Jan 10, 2025. Publication status criteria included: matching at least three keywords between the abstract and any resulting manuscript, having at least one common author, and publication occurring after and inclusive of the year of abstract acceptance. Data was compiled into an Excel spreadsheet, categorizing abstracts as ‘yes’ or ‘no’ for publication. Publication rates were then calculated using Excel formulas based on these categorizations. Factors associated with publication were evaluated, and publication metrics were described. **Result:** All 351 abstracts were analyzed. Among these, 175 (49.9%) were published as full articles in peer-reviewed journals indexed in Google Scholar or PubMed. Abstracts presented in 2021 and those presented orally had higher publication rates, though the association was statistically nonsignificant (p = 0.06 and p = 0.66, respectively). Abstracts with authors from different institutions and those with more than six authors showed a statistically significant association with higher publication rates (p = 0.002 and p = 0.003, respectively). Infection Control & Hospital Epidemiology was the most common journal in which abstracts were ultimately published, accounting for 51 (29.1%) of the publications. The publication rates surpass those reported in most similar studies of other internal medicine and subspecialty conferences, including IDWeek. **Conclusion:** Approximately half of the abstracts presented were subsequently published as full articles. Collaborative research, involving more authors and authors from different institutions, was associated with a higher publication rate. These findings highlight the strong academic impact of SHEA-presented research. Further research into the barriers to publication is warranted to improve the dissemination of conference abstracts.

**Figure:**
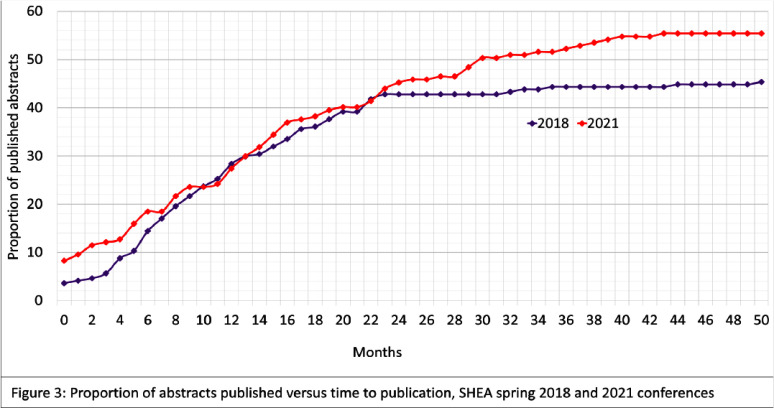

**Figure:**
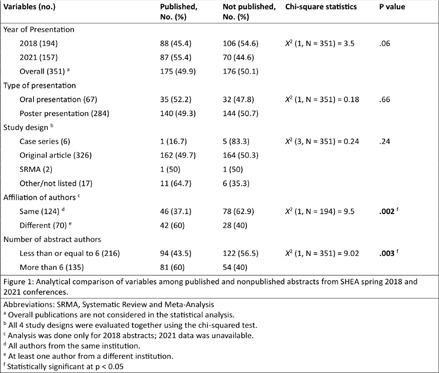

**Figure:**
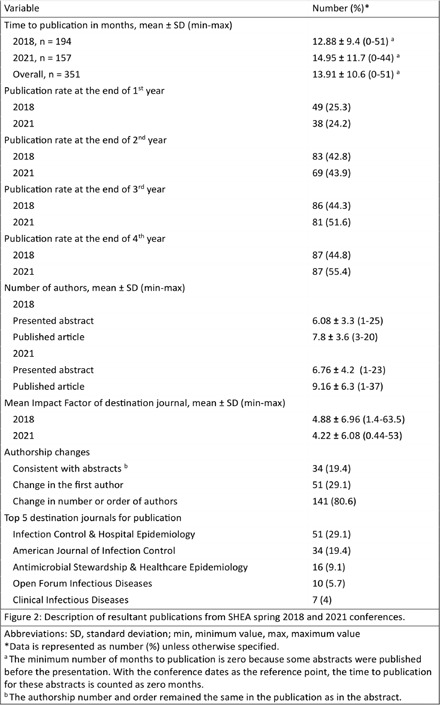

**Figure:**